# Low Cost, Easily-Assembled Centrifugal Buoyancy-Based Emulsification and Digital PCR

**DOI:** 10.3390/mi13020171

**Published:** 2022-01-24

**Authors:** Wuping Zhou, Cong Liu, Tao Zhang, Keming Jiang, Haiwen Li, Zhiqiang Zhang, Yuguo Tang

**Affiliations:** 1 Division of Life Sciences and Medicine, School of Biomedical Engineering (Suzhou), University of Science and Technology of China, Hefei 230026, China; zhouwp@sibet.ac.cn; 2 Key Lab of Bio-Medical Diagnostics, Suzhou Institute of Biomedical Engineering and Technology, Chinese Academy of Sciences, Suzhou 215163, China; liuc@sibet.ac.cn (C.L.); zhangtao@sibet.ac.cn (T.Z.); jiangkm@sibet.ac.cn (K.J.); zhangzq@sibet.ac.cn (Z.Z.)

**Keywords:** centrifugal droplet generation, buoyancy, easily-assembled, digital polymerase chain reaction (dPCR), K-RAS mutation

## Abstract

Microfluidic-based droplet generation approaches require the design of microfluidic chips and a precise lithography process, which require skilled technicians and a long manufacturing time. Here we developed a centrifugal buoyancy-based emulsification (CBbE) method for producing droplets with high efficiency and minimal fabrication time. Our approach is to fabricate a droplet generation module that can be easily assembled using syringe needles and PCR tubes. With this module and a common centrifuge, high-throughput droplet generation with controllable droplet size could be realized in a few minutes. Experiments showed that the droplet diameter depended mainly on centrifugal speed, and droplets with controllable diameter from 206 to 158 μm could be generated under a centrifugal acceleration range from 14 to 171.9 *g*. Excellent droplet uniformity was achieved (CV < 3%) when centrifugal acceleration was greater than 108 *g*. We performed digital PCR tests through the CBbE approach and demonstrated that this cost-effective method not only eliminates the usage of complex microfluidic devices and control systems but also greatly suppresses the loss of materials and cross-contamination. CBbE-enabled droplet generation combines both easiness and robustness, and breaks the technical challenges by using conventional lab equipment and supplies.

## 1. Introduction

Homogenous water-in-oil emulsion has become increasingly essential to many biomedical applications, such as isolation of cells or enzymes [1,2,3], synthetization of micro-particles [4,5], and digital PCR or digital LAMP [6,7]. In the past decades, microfluidic-based droplet generation approaches have been proved to be robust and efficient methods that can produce uniform droplets with polydispersity lower than 10% [8,9].

Microfluidic-based droplet generation approaches offer many advantages, such as good uniformity, controllable droplet size, and controllable producing rate. The common microfluidic approaches for droplet emulsification are step emulsification [10,11], T-junction [12,13], and flow-focusing [14,15] systems. During these approaches, sophisticated pumps [16,17] or pressure sources [14,18], together with tubings, are used to control the flow rate of the dispersed phase, the continuous phase, or both, to flow through fine-patterned micro-channels and droplet generation microstructures, to achieve a narrow distribution of droplet volumes.

Even though droplet microfluidic technology has made great progress; however, it still lags behind the high-throughput and high-efficiency droplet preparation required by researchers, mainly including: First, the long pipeline connection between the pump and the chip increases the loss of precious samples and reagents, as well as the difficulty of removing bubbles in the flow channel. Second, skilled workers are required for installing, adjusting, and operating these microfluidic systems.

Lab-on-a-disc (LOD) technology overcomes these shortcomings of conventional microfluidic technology by using centrifugal force instead of traditional pumps to pump fluid. Step emulsification [6,7,19,20,21,22] and crossflow [23,24,25,26] are commonly used in lab-on-a-disc (LOD) devices. Schuler et al. developed a high-throughput droplet preparation method by using centrifugal step emulsification technology. They carried out droplet digital RPA [27] and digital LAMP [28]. Schuler et al. also studied the effect of centrifugal buoyancy on the preparation of droplets by step emulsification [22]. Shin et al. developed a small step emulsification module using polydimethylsiloxane (PDMS) [21]. They placed this module in a micro-tube, and generated pico-liter droplets using a centrifuge. Clime et al. introduced a pneumatic source into centrifugal step emulsification, and studied the interplay between buoyancy effects and the flow rate at the step junction [20]. Madadelahi et al. developed a structure with a fluidic barrier strategy for generating multiple emulsions and microspheres [29]. They skillfully formed a structure similar to a dispenser nozzle through multi-layer substrates with microstructure. The sample is detached by centrifugal force into droplets, and then flowed through a fluid barriers structure to form multiple emulsions microsphere.

Even though LOD-based droplet microfluidic technology has been widely studied, long manufacturing time is still needed for processing microfluidic chips using traditional soft-lithography process [12,14,15] or precision machining [27,29,30].

Droplet generation methods which are independent of precision machining and sophisticated pumps, and using finished products existing in a conventional laboratory have become a research hotspot [31,32,33,34,35]. Among these methods, the dispenser nozzle-based droplet generation method, which is composed of micro-needles, centrifugal tubes, and a centrifuge, has been widely studied [31,32,36,37,38]. Takeuchi et al. have reported the centrifuge-based droplet shooting device (CDSD) for the generation of 3D multi-compartmental particles using a multi-barreled capillary [37]. Previously, Morita et al. developed a droplet-shooting and size-filtration (DSSF) method for the synthesis of cell-sized liposomes with controlled lipid compositions [32].

Although the centrifuge-based droplet shooting system has successfully been presented to generate the complex droplets, these techniques still have many drawbacks, including droplet loss due to collision between the high-speed droplet and surface of the oil phase, and the density of the oil phase must be less than that of sample phase.

**Figure 1:**
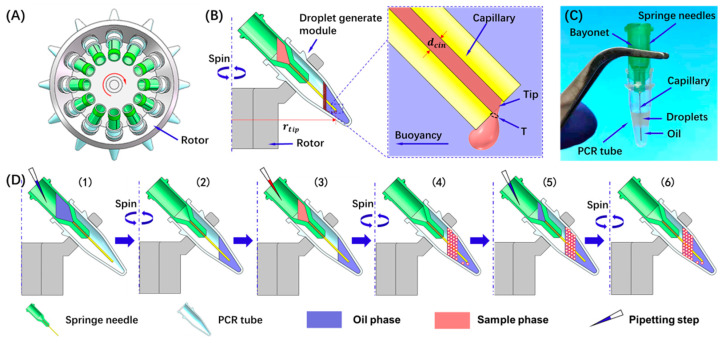
System description and workflow of the CBbE method. (**A**) Droplet generation using a benchtop centrifuge, needle, and PCR tube. (**B**) Working principle of the CBbE method. When the sample is transferred through the capillary and flows out of the tip, the buoyancy force causes the extruded sample to break off into a droplet. (**C**) Needle and PCR tube with droplets generated by the CBbE method. (**D**) Workflow of the CBbE emulsification with zero dead volume. Step 1: Add oil to the needle. Step 2: Spinning forces oil to flow through the capillary and into the PCR tube, causing the tip of the capillary to become hydrophobic. Step 3: Aqueous sample is added to the needle. Step 4: The sample is emulsified during centrifugation, while some sample remains in the capillary. Step 5: Add oil into the needle again. Step 6: Spinning forces more oil into the capillary, which pushes the remaining sample out to form more droplets.

Herein, we present a novel method for generating digital PCR droplets using a centrifugal buoyancy-based emulsification (CBbE) approach. The CBbE system is illustrated in Figure 1. The whole system contains a centrifuge and an assembly of a springe needle and a PCR tube. Unlike the CDSD approach whose capillary tip is located above the oil phase [31,32], the tip of the needle used in our method is located in the oil phase, and the buoyancy is the dominant force in the formation of droplets. Upon spinning, when the sample phase flows out of the needle’s tip, it is detached from the needle under centrifugation-induced buoyancy to form a droplet in the oil phase. The speed of droplets was slowed down due to the drag force from the oil phase, therefore high-speed collision of droplet and oil phase surface was reduced. This strategy is very attractive in that the emulsification system preparation and operation are very easy and convenient, requiring no complicated skill but just a centrifuge and an easily assemble module. In addition, this strategy is not only useful to generate droplets but can also yield scalable production.

Digital droplet PCR was performed using the CBbE method for K-RAS mutation. K-RAS mutation plays a critical role in the outcome and prognosis of many cancers, such as colon and pancreatic cancer. Thus, its early detection is very important.

## 2. Materials and Methods

The construction and operation principle of the CBbE approach is shown in Figure 1. The CBbE system consisted of a centrifuge and an assembly of a 34 G springe needle (Jinrong, Shanghai, China) and a 200 μL PCR tube (Axygen, NY, USA). The spring needle was inserted into the PCR tube, and a tight fit was formed as the outer wall of the needle is slightly bigger than the inner wall of the PCR tube. The distance between the needle’s tip and the bottom of the PCR tube $d_{tb}$ was kept at about 1.5 mm. The needle contains a stainless steel capillary with an inner diameter $d_{cin}$ of about 60 μm and an outer diameter $d_{cout}$ of about 250 μm. The inner diameter of the capillary was measured using a measuring microscope (HISOMET II, Union Optical, Tokyo, Japan) before each experiment. Under the infiltration of the oil phase, the contact angle between the capillary surface and the sample phase is 141° (see Supplementary Materials Figure S1). A bench-top centrifuge (NS-12K, ENYI, Changzhou, China) with adjustable rotation speed from 0 to 12,000 rpm was purchased from Taobao, and its rotating speed was calibrated by a digital tachometer (MS6208B, Peakmeter, Shenzhen, China). In all experiments, droplet generation oil for PCR probes (#D9161172A) and ddPCR supermix for probes (#1863026) were purchased from Bio-Rad, USA. Geometry dimensions and material properties can be found in Supplementary Materials Table S1.

The fabricated emulsion was injected into a custom-made droplet observation chamber (See Supplementary Materials Figure S2) for imaging. Images were obtained using a microscope (BX53, OLYMPUS, Tokyo, Japan) and processed by ImageJ V1.8 (See Supplementary Materials Figure S5). Mean and standard deviation of droplet diameters $d_{drop}$ were obtained by statistical analysis of 10 different regions, with each region containing more than 100 droplets. The droplet observation chamber was micro-milled in polymethylmethacrylate (Shenzhen Anheda Plastic, Shenzhen, China) to a depth of 200 μm using a CNC machine (VF2SS, Haas Automation, Oxnard, CA, USA), and then sealed by a single-sided adhesive tape (90697, Adhesives Research, Inc., Glen Rock, PA, USA).

For the digital PCR assay of K-RAS mutation, the whole genome sequence of the K-RAS gene was obtained from the NCBI GENE BANK database (NG 007524), and Premier 5.0 software was used to design the primer and probe for amplification of the No. 12 codon in the selected sequence area. All primers and probes were synthesized by Invitrogen Co., Ltd. (Shanghai, China) as follows:

Forward primer: CCCAGCAACAGCACAACCC

Reverse primer: GCCGCAGCGTAACTATTACTAATG

Wild type gene probe: 5′-HEX-ttggagagctggtggcgt-MGB

Mutant probe: 5′-FAM-tggagctgatggcgt-MGB

An enzyme site sequence was added at both ends of the No. 12 codon of the K-RAS gene, which also contained a G12D mutation. Both corresponding gene sequences were synthesized by Suzhou Jinweizhi Biotechnology, Suzhou, China, and then connected with a pUC57 plasmid after enzyme cutting to construct the wild type and G12D mutant. The mutated K-RAS plasmid was cloned, transformed, and verified by electrophoresis and sequencing of the enzyme cuts.

Each PCR mixture contains 20 μL PCR Mix, 1 μL forward primer, 1 μL reverse primer, 1 μL probe, 15 μL ultrapure water, and 2 μL K-RAS sample. Ultrapure water was used instead of the sample as NTC (no template control).

After PCR cycling using a traditional PCR-Cycler (Mini3210, LongGene, Hangzhou, China), the emulsions were injected into the droplet observation chamber for fluorescence imaging using a microscope (BX53, OLYMPUS, Tokyo, Japan) with 475 nm excitation and 530 nm emission wavelengths. ImageJ V1.8 was used for the evaluation of the images. Negative and positive droplet numbers were obtained by droplet’s fluorescence intensity with a threshold defined manually. The DNA concentration *C* of the initial sample was obtained using the negative and positive droplet numbers based on Poisson distribution.

(1) $$C=-\frac{ln\left(\frac{n^{-}}{n^{-}+n^{+}}\right)}{V_{drop}}$$

where $n^{-}$ and $n^{+}$ are the negative and positive droplet numbers, and $V_{drop}$ is the droplet volume.

## 3. Results and Discussion

### 3.1. Working Principle

As shown in Figure 1 and Figure S4, when the sample phase flows out of the capillary’s tip under spinning, there are primarily two forces—centrifugal force-induced buoyancy and interfacial tension—acting on the drops. As the density of the oil phase (fluoride oil, 1.8 × 10^3^ kg/m^3^) is greater than the discrete phase (PCR mixture, 1.0 × 10^3^ kg/m^3^), the buoyancy is toward the oil phase surface, and is proportional to the volume of the sample out of the tip, while the interfacial tension keeps almost constant (Figure 1B). When buoyancy is greater than the surface tension, the sample is detached from the tip to form droplets and flows toward the oil surface. After being detached, there are primarily two forces—centrifugal force-induced buoyancy and drag force—acting on the drops. The buoyancy can be expressed as

(2) $$F_{b}=\Delta \rho gV_{drop}$$

where Δρ is the density difference between the sample phase and oil phase and $V_{drop}$ is the volume of the drop. The drag force can be estimated by the Stoke’s law as follows.

(3) $$F_{d}=3\pi \eta_{oil}d_{drop}v$$

where $\eta_{oil}$ is the viscosity of the oil, $d_{drop}$ is the drop diameter, and v is the relative velocity between the drop and the stagnant oil phase. The maximum velocity of the droplets is obtained when the buoyancy and drag forces are balanced. This is quite different from existing technology [32,39], which detaches the sample from the capillary’s tip into droplets by centrifugal-induced gravity. In their study, droplets crash to the oil surface at a high speed after flying through a section of air. Our approach is to generate droplets under the action of buoyancy and move slowly toward the oil phase surface due to the drag force from the oil phase. The drag force is proportional to the droplet’s moving velocity, which can effectively slow down the droplet. Thus, fragmentation and fusion of droplets caused by the collision between droplets and oil surface are reduced. In addition, the PCR tube used in this method is compatible with the existing PCR amplification instrument, which reduces pipetting steps.

### 3.2. Generation of Droplets

**Figure 2:**
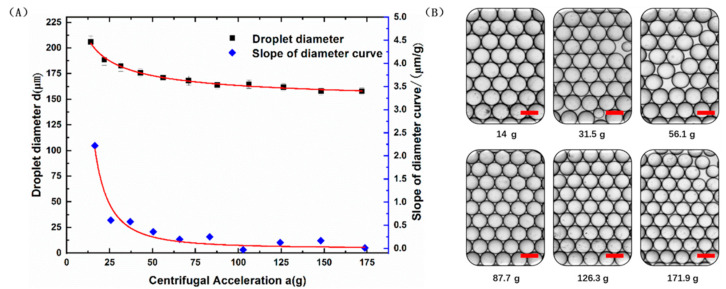
Experiments with different centrifugal accelerations using the CBbE method. (**A**) Experiments with different centrifugal accelerations from 14 to 171.9 *g* were studied, with the capillary inner diameter and tip centrifugal radius held constant ($d_{cin}$ = 60 μm, $r_{tip}$ = 20 mm). The slope of the diameter curve was calculated as ∆d/∆a. Each data point corresponds to three independent measurements of droplet diameter. Error bars given are standard deviations (SD). (**B**) Microscope images of the produced droplets using different centrifugal accelerations. Scale bars = 200 μm.

Droplet diameter as a function of centrifugal acceleration was shown in Figure 2A. Droplets with diameter $d_{drop}$ of 206 to 158 μm were generated under a centrifugal acceleration range of 14 to 171.9 *g*. Droplet diameter decreased gradually while increasing centrifugal acceleration. In addition, the descent rate of droplet diameter also decreased while increasing centrifugal acceleration (see the slope of diameter curve in Figure 2A). This was a very attractive result, indicating that droplet size became less sensitive to the deviation in centrifugal acceleration. When centrifugal acceleration was greater than 108 *g*, the coefficient of variation (CV) of droplet diameter was evaluated and found to be <3%, showing very good robustness.

**Figure 3:**
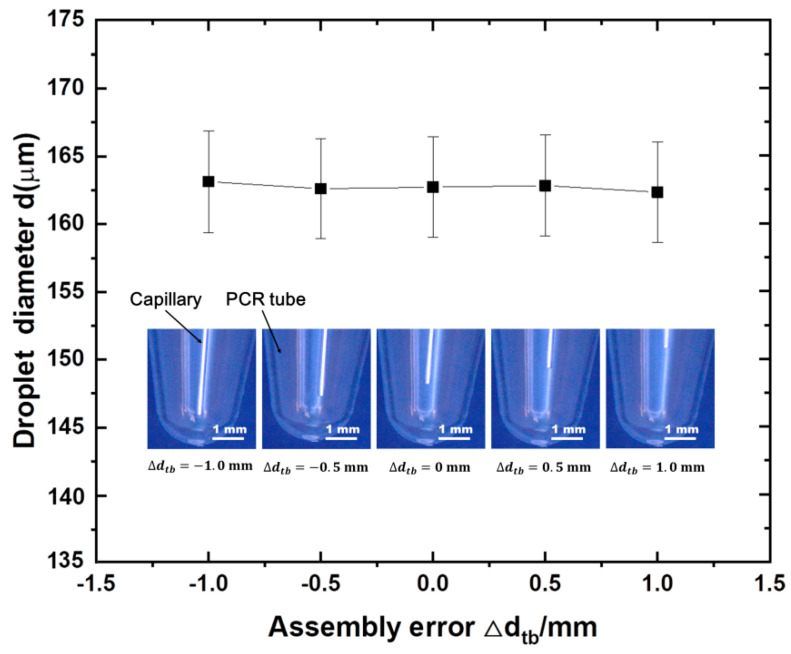
Experiments with different assembly errors. Experiments were carried out when centrifugal acceleration was held constant (108 *g*). Each data point corresponds to three independent measurements of droplet diameter. Error bars given are standard deviations (SDs).

As the assembly of the droplet generating module was designed to be easy to use, we sought to avoid requirements for precise alignment or positioning. In addition to the centrifugal acceleration, the assembly error of the droplet generation module and the inner diameter of the needle’s capillary will affect the droplet size too. Assembly error, $∆d_{tb}$, is defined as the deviation of $d_{tb}$ from its ideal position $d_{tb0}$, see Supplementary Materials Figure S3. Droplet sizes with $∆d_{tb}$ of −1, −0.5, 0, 0.5, and 1.0 mm were generated (see Figure 3). It can be seen that the assembly error has little effect on the prepared droplets, showing excellent robustness.

**Figure 4:**
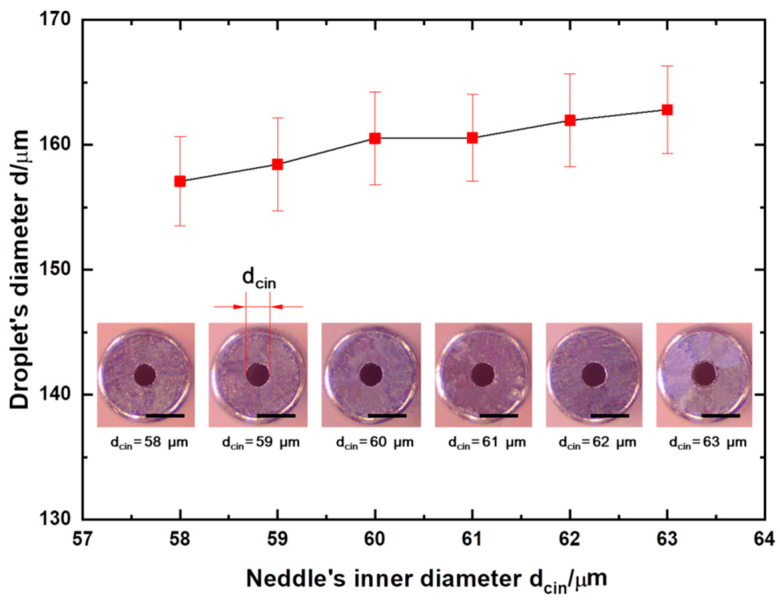
Experiments with different capillary inner diameters. Experiments were carried out when centrifugal acceleration $a_{tip}$ was held constant (108 g). Error bars given are standard deviations (SDs). Scale bars: 100 μm.

To estimate the effect of capillary inner diameter $d_{cin}$ on droplet size, six needles with different inner diameters were selected from a batch of springe needles. On the premise of ensuring the assembly error as little as possible, droplet preparation was carried out (See Figure 4). In general, a larger needle’s diameter resulted in a larger droplet size. When the needle inner diameter increased from 58 to 63 μm, the mean droplet size increased from 157 to 162 μm, with a relative change of about 3%, indicating that the capillary inner diameter has little effect on the preparation of droplets.

### 3.3. Digital PCR

**Figure 5:**
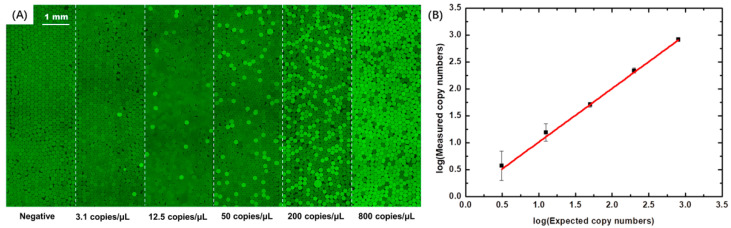
Digital PCR performed for K-RAS using the CBbE approach. (**A**) Single-layer droplet fluorescence images of droplet-based digital PCR. Dark green indicates negative droplets and light green indicates positive droplets. (**B**) Comparison of measured and expected copy numbers. The measured copy numbers were obtained by analysis of more than 5000 droplets using Poisson statistics, with each data point corresponding to three independent digital PCR measurements. The expected copy numbers were estimated from qPCR measurements. The solid red line is a linear fit (R^2^ > 99.2%). Error bars given are standard deviations (SD).

For robust digital PCR, a centrifugal acceleration of 108 g was selected, indicating a droplet diameter of 158 μm, and a CV of less than 3%. Stock K-RAS template solution with a concentration of 800 copies/µL, calibrated by qPCR (ABI7500, ThermoFisher, Waltham, MA, USA), was serially diluted to 200, 50, 12.5, and 3.1 copies/µL. Together with NTC, digital PCR was performed on six samples (Figure 5). The correlation coefficient between the experimental and expected results was 0.992, showing that this system can accurately quantitate the amount of DNA, which could be detected at concentrations as low as 3.1 copies/µL where the relative error was about 27%.

## 4. Conclusions

We presented a novel CBbE method for droplet preparation characterized by high efficiency, low cost, and easily adjusted droplet volume. The structure used here is easy to use (two to three pipetting steps required) and works without any pumps or tubing. This approach was composed of a PCR tube, a springe needle, and a bench-top centrifuge. Droplets with controllable diameter from 206 to 158 μm were generated under a centrifugal acceleration range of 14 to 171.9 *g*. Excellent droplet uniformity was achieved (CV < 3%) when centrifugal acceleration is greater than 108 *g*. Droplet diameter was insensitive to the micro-needle inner diameter’s deviation and assembly error, showing excellent robustness. Digital PCR of K-RAS mutation was carried out using the CBbE method, and a strong correlation (0.992) was observed between the measured and expected copy numbers, indicating that the CBbE method has great potential in the field of digital PCR.

The main disadvantage of the CBbE method is that the droplet size cannot be further decreased by increasing centrifugal acceleration. Because, when increasing centrifugal acceleration, the capillary number and bond number also increase, leading to an unstable jetting regime.

This study has some innovative points compared to previous dispenser nozzle-based researches [29,31,36,37,38,39,40]: (1) Centrifugal-induced buoyancy, rather than centrifugal-induced gravity, is adopted and plays a leading role in droplet formation. (2) An angle of 135° exists between the movement of the sample in the capillary and the movement of the prepared droplets. This angle ensures that the initial droplet velocity is zero. Additionally, compared to existing dispenser nozzle systems, a smaller droplet-generating module size can be achieved.

An additional advantage of this method lies in its unique centrifugal drive and ultra-small droplet preparation module, which allows for the integration of micro-size and ultra-high throughput droplet preparation. Therefore, we believe that the CBbE method will create considerable progress, not only in droplet digital PCR fields, but also in the production of monodisperse functional particles and the isolation of cells or enzymes.

## Data Availability

Not applicable.

## Supplementary Materials

The following supporting information can be downloaded at: https://www.mdpi.com/article/10.3390/mi13020171/s1, Figure S1: Contact angle between the needle’s capillary surface and the sample phase in the oil; Figure S2: Droplet observation chamber; Figure S3: Detailed definition of the assembly error $∆d_{tb}$; Figure S4: Cross-section view of the CBbE method; Figure S5: Image process using imageJ v1.80; Table S1: Geometry dimensions and material properties of the CBbE method; Table S2: Calculation of centrifugal acceleration at the capillary’s tip and sample’s flow rate in capillary.
