# Supplementary material for: Low Cost, Easily-Assembled Centrifugal Buoyancy-Based Emulsification and Digital PCR
Source: Micromachines (Basel). 2022 Jan 24;13(2):171. doi: 10.3390/mi13020171 (PMC8924881; doi:10.3390/mi13020171)
Supplement: Supplementary file 1 [file micromachines-13-00171-s001.zip › micromachines-1543646 - Supplementary Information - corrected.pdf]

micromachines

Supplementary Information (SI)

Low Cost, Easily-assembled Centrifugal Buoyancy-based Emulsification and Digital PCR

Wuping Zhou[1,2], Cong Liu[2], Tao Zhang[2], Keming Jiang[2], Haiwen Li[2,*], Zhiqiang Zhang[2], Yuguo Tang[2,*]

[1] School of Biomedical Engineering (Suzhou), Division of Life Sciences and Medicine, University of Science and Technology of China, Hefei 230026, China; zhouwp@sibet.ac.cn

[2] Key LAB of Bio-Medical Diagnostics, Suzhou Institute of Biomedical Engineering and Technology, Chinese Academy of Science, Suzhou 215163, China; liuc@sibet.ac.cn (C.L.); zhangtao@sibet.ac.cn (T.Z.);jiangkm@sibet.ac.cn (K.J.); zhangzq@sibet.ac.cn (Z.Z.)

* Correspondence: lihw@sibet.ac.cn (H.L.); tangyg@sibet.ac.cn (Y.T.); Tel.: +86-188-6211-3320 (H.L.)

1. Figures S1-S3.

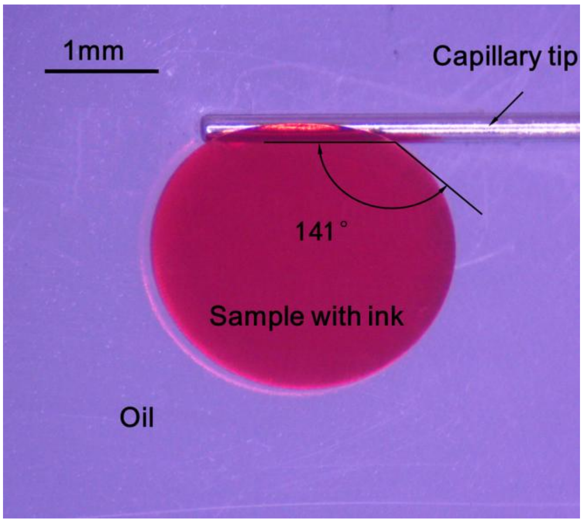

Figure S1: Contact angle between the needle's capillary surface and the sample phase in the oil.

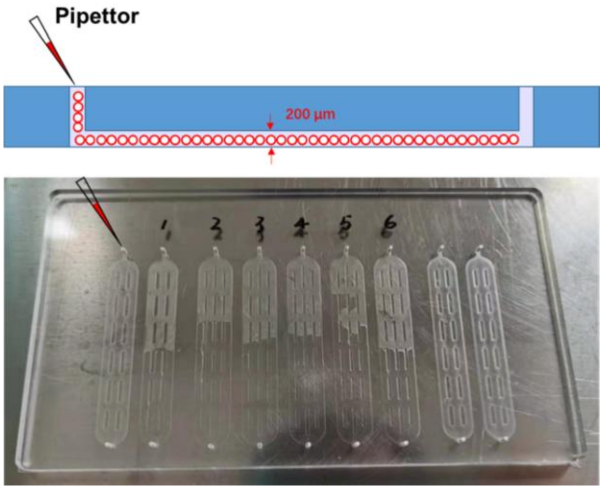

Figure S2. Droplet observation chamber.

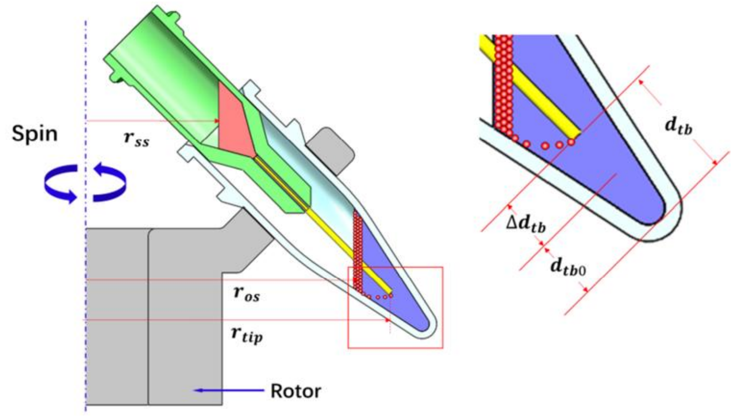

Figure S3. Detailed definition of the assembly error Δd_{tb}.

2. Detailed view of the cross-section of CBbE approach.

A cross-section view of the CBbE method is displayed in Figure S4, Geometry dimensions and material properties are listed in Table S1.

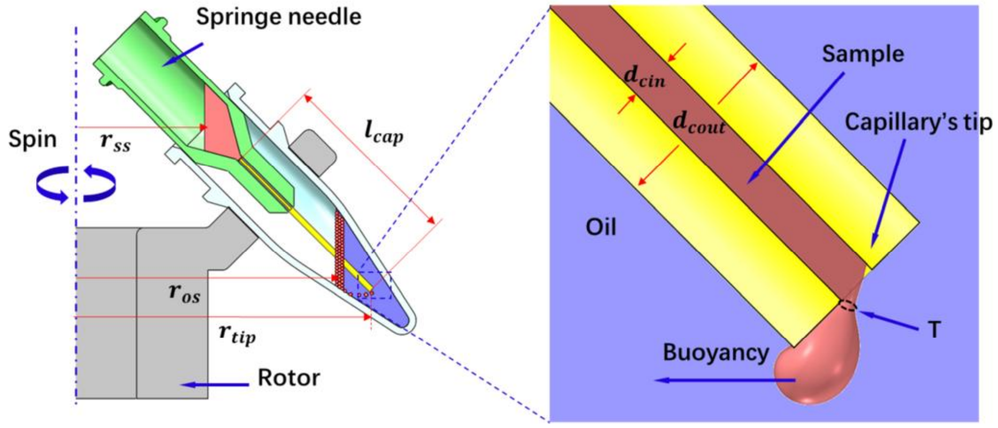

Figure S4. Cross-section view of the CBbE method.

Table S1. Geometry dimensions and material properties of the CBbE method.

Abbreviation	Description	Start value	End value
r_{ss}	Centrifugal radius of sample surface	7 mm	9 mm
r_{os}	Centrifugal radius of oil surface	19 mm	17 mm
r_{tip}	Centrifugal radius of the tip	20 mm	
d_{cin}	Diameter of capillary's inner wall	0.06 mm	
d_{cout}	Diameter of capillary's outer wall	0.25 mm	
l_{cap}	Length of capillary	20 mm	
ρ_{sam}	Density of sample phase	1.0×10^{3} Kg/m^{3}	
ρ_{oil}	Density of oil phase	1.8×10^{3} Kg/m^{3}	
μ_{sam}	dynamic viscosity of sample phase	1.0×10^{-3} Pa•s	

3. Calculation of centrifugal acceleration at the tip

When the centrifuge is working at a rotating speed n, angular velocity ω can be calculated by:

$$\omega = \frac{2\pi n}{60} \tag{S1}$$

Centrifugal acceleration at the tip is given by:

$$a_{tip} = \omega^2 r_{tip} \tag{S2}$$

4. Calculation of sample's flow rate q in capillary.

Pressure drop from r_{ss} to r_{tip} in sample phase is given by:

$$\Delta p_{ss-tip} = \frac{1}{2}\rho_{sam}\omega^2\left({r_{tip}}^2 - {r_{ss}}^2\right) \tag{S3}$$

Pressure drop from r_{os} to r_{tip} in oil phase is given by:

$$\Delta p_{os-tip} = \frac{1}{2}\rho_{oil}\omega^2\left({r_{tip}}^2 - {r_{os}}^2\right) \tag{S4}$$

Actuating pressure can be calculated by:

$$\Delta p = \Delta p_{ss-tip} - \Delta p_{os-tip} \tag{S5}$$

Under the assumption of laminar flow in the needle's capillary, hydraulic resistance R_{hyd} can be calculated by:

$$R_{hyd} = \frac{128\mu_{sam}l_{cap}}{\pi {d_{cin}}^4} \tag{S6}$$

Thus, the sample's flow rate q in the capillary can be obtained by:

$$q = \frac{\pi {d_{cin}}^4}{128\mu_{sam}l_{cap}}\Delta p \tag{S7}$$

Let $r_{ss} = 8\,mm$ and $r_{os} = 18mm$, flow rate q with different centrifugal acceleration a_{tip} can be calculated, see Table S2.

Table S2. Calculation of centrifugal acceleration at the capillary's tip and sample's flow rate in capillary.

rotating speed n (rpm)	a_{tip} (g)	q (μl/min)
600	8.056806	0.375218
800	14.32321	0.667055
1000	22.38002	1.042273
1200	32.22723	1.500874
1400	43.86483	2.042856
1600	57.29284	2.66822
1800	72.51126	3.376966
2000	89.52007	4.169093
2200	108.3193	5.044603
2400	128.9089	6.003494
2600	151.2889	7.045768
2800	175.4593	8.171423

5. Detailed description of the droplet diameter measurement.

The fabricated emulsion was injected into a custom-made droplet observation chamber for imaging. The microscope camera used in the experiment is the alA1920-40 camera (Basler Vision Technology, Ahrensburg, Germany) and its pixel is square with its side length l_p of 5.86 μm. When imaging with a 4x objective lens, the actual side length l_r of each pixel in the picture can be obtained by:

$$l_r = l_p/4 \tag{S8}$$

The droplet diameter was measured as described as listed in the following section (see also Fig.S5):

1). The stroboscopic image was imported into the image processing software ImageJ v1.80.

2). The edge of the droplet is recognized, and the number of pixels A_p occupied by the droplet area is measured. So droplet diameter in pixels can be obtained by:

$$d_p = 2\sqrt{\frac{A_p}{\pi}} \tag{S9}$$

3). The actual diameter d_r of the measured droplets can be obtained by equation (S8):

$$d_r = d_p \times l_r \tag{S10}$$

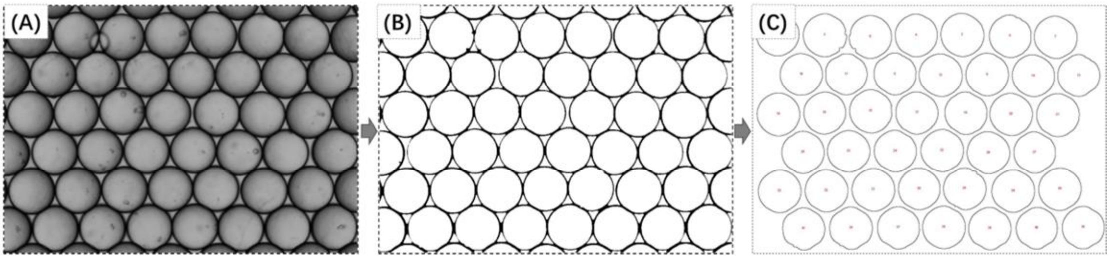

Figure S5. Image process using imageJ v1.80. (A) Original image. (B) Droplet edge recognized by imageJ. (C) Droplet area recognized by imageJ.

6. Calculation of DNA concentration of the initial sample using Poisson distribution..

Under the assumption that the total amount of target molecules (m) is distributed throughout the generated droplets (n) by the Poisson-distribution [1], the probability that a droplet will contain k copies of the targets can be calculated by:

$$p(\mathrm{k}) = \frac{\lambda^{\mathrm{k}} \mathrm{e}^{-\lambda}}{\mathrm{k}!} \tag{S11}$$

Where, λ is the mean occupancy rate and is given by

$$\lambda = \frac{m}{n} \tag{S12}$$

Then the probability that a droplet is a negative one is:

$$p(0) = \frac{\lambda^0 \mathrm{e}^{-\lambda}}{0!} = \mathrm{e}^{-\lambda} = \frac{n^-}{n} = \frac{n^-}{n^- + n^+} \tag{S13}$$

Where, n^- and n^+ are the negative and positive droplet numbers. Therefore, the mean occupancy rate (λ) can be calculated from equation (S13):

$$\lambda = -\ln\left(\frac{n^-}{n^- + n^+}\right) \tag{S14}$$

The DNA concentration C f the initial sample can be obtained by:

$$C = \frac{\lambda}{V_{drop}} = \frac{\ln\left(\frac{n^-}{n^- + n^+}\right)}{V_{drop}} \tag{S15}$$

References

1. Basu, Amar S. (2017): Digital Assays Part I: Partitioning Statistics and Digital PCR. In: SLAS technology 22 (4), S. 369–386. DOI: 10.1177/2472630317705680.
